# Strategies for price reduction of HIV medicines under a monopoly situation in Brazil

**DOI:** 10.1590/S0034-8910.2015049005459

**Published:** 2016-03-01

**Authors:** Gabriela Costa Chaves, Lia Hasenclever, Claudia Garcia Serpa Osorio-de-Castro, Maria Auxiliadora Oliveira

**Affiliations:** I Departamento de Política de Medicamento e Assistência Farmacêutica. Escola Nacional de Saúde Pública Sergio Arouca. Fundação Oswaldo Cruz. Rio de Janeiro, RJ, Brasil; II Grupo de Economia da Inovação. Instituto de Economia. Universidade Federal do Rio de Janeiro. Rio de Janeiro, RJ, Brasil

**Keywords:** Anti-HIV Agents, supply & distribution, Drug Price, Health Care Costs, Intellectual Property, National Drug Policy

## Abstract

**OBJECTIVE:**

To analyze Government strategies for reducing prices of antiretroviral medicines for HIV in Brazil.

**METHODS:**

Analysis of Ministry of Health purchases of antiretroviral medicines, from 2005 to 2013. Expenditures and costs of the treatment per year were analyzed and compared to international prices of atazanavir. Price reductions were estimated based on the terms of a voluntary license of patent rights and technology transfer in the Partnership for Productive Development Agreement for atazanavir.

**RESULTS:**

Atazanavir, a patented medicine, represented a significant share of the expenditures on antiretrovirals purchased from the private sector. Prices in Brazil were higher than international references, and no evidence was found of a relationship between purchase volume and price paid by the Ministry of Health. Concerning the latest strategy to reduce prices, involving local production of the 200 mg capsule, the price reduction was greater than the estimated reduction. As for the 300 mg capsule, the amounts paid in the first two years after the Partnership for Productive Development Agreement were close to the estimated values. Prices in nominal values for both dosage forms remained virtually constant between 2011 (the signature of the Partnership for Productive Development Agreement), 2012 and 2013 (after the establishment of the Partnership).

**CONCLUSIONS:**

Price reduction of medicines is complex in limited-competition environments. The use of a Partnership for Productive Development Agreement as a strategy to increase the capacity of local production and to reduce prices raises issues regarding its effectiveness in reducing prices and to overcome patent barriers. Investments in research and development that can stimulate technological accumulation should be considered by the Government to strengthen its bargaining power to negotiate medicines prices under a monopoly situation.

## INTRODUCTION

Since 1996, Brazil ensures free universal access to antiretroviral medicines (ARV) and other medicines necessary for the treatment and control of HIV infection, through the Brazilian Unified Health System (SUS).^7^ Over the years, prices, costs and increasing expenditure on medicines under a monopoly situation threaten the financial sustainability of the Brazilian response to the epidemic.^7,18,20^

The expenses of the Ministry of Health (MoH) have increased due to: the increasing number of people living with HIV receiving ART (antiretroviral therapy); the emergence of viral strains resistant to first and second line regimens; and to the incorporation of new ARV.^6,11^ The need to migrate to second and third-line therapeutic regimens, due to the emergence of viral resistance, requires the use of more expensive ARV, usually imported and under a monopoly situation.^7^

In the current situation, of the implementation of the Agreement on Trade-Related Aspects of Intellectual Property Rights (TRIPS Agreement) of the World Trade Organization (WTO), the newest ARV are under a monopoly situation in Brazil and internationally. This increases the difficulty to negotiate and obtain price reductions.^3,11,18^

The ARV medicines under a monopoly situation are those offered by a single supplier, generally because they are subject to patent protection (patent application filed or patent granted in the country).

Studies have examined the evolution of the Ministry of Health expenditure on ARV and the determinants of prices in a historical series from 1996 to 2009.^6,9-11,16,a^ For ARV under a monopoly situation their prices reduced initially, but this gain was lost over time. A significant increase of expenditure on patented ARV was observed, which reached 80.0% of MoH resources allocated for the purchase of all ARV in 2004 and 2005.^16^

Medicines that have multiple suppliers are more sensitive to variables such as purchase volumes. The prices of medicines under a monopoly situation are less sensitive to purchase volumes, but are affected when the MoH bargaining power in price negotiations is strengthened by the use of: evidence on production costs; the threat or issuance of a compulsory license, amongst others.^16^ Bargaining power is reduced when the local technological and industrial capacity or the alternatives suppliers do not exist or are limited.^9,11,16^

International initiatives to tackle ARV prices under a monopoly situation were implemented in developing countries in the 2000s. Major donors, such as the Global Fund to Fight AIDS, Tuberculosis and Malaria and the Unitaid, had an important role in market dynamics and price reductions by purchasing large quantities and using market guarantees to stimulate the development of fixed-dose combinations and pediatric formulations.^25^

Multinational pharmaceutical companies, in turn, have adopted price discrimination policies (“tiered pricing”), creating categories of price discounts based on criteria established by them, which can include country’s level of development and/or the national prevalence of HIV.^13,24^ However, the same criteria are not universally adopted by companies, and countries might be eligible to price discounts offered by some companies but not by others.

One recent initiative that aims to overcome patent barriers and to encourage the availability of fixed-dose combinations and pediatric formulations is the creation of the Medicines Patent Pool (MPP), which negotiates voluntary licenses with multinational pharmaceutical companies to promote generic competition and reduce prices.^b^

Although these international initiatives show results in the expansion of access to ARV and addressing some high prices, Brazil is excluded from all of them and has had to develop its own strategies.

The Brazilian Government’s main strategies to reduce ARV prices under a monopoly situation, from 2001 to 2007, included: price negotiation with multinational pharmaceutical companies, with the threat of issuing compulsory licenses, based on estimates of production cost^19^ and international reference prices; the challenging of patent applications by a public manufacturer;^1^ the issuance of a compulsory license for importation and subsequent local production of the medicine.^11,22^

The study of governmental strategies to ensure access to ARV in a country like Brazil includes understanding the process of technology incorporation in the health system, approaches to ensure availability (regarding the maintenance and expansion of treatment) as well as initiatives for tackling prices of products under a monopoly situation, including efforts for local production.

The objective of this study was to analyze Government strategies to reduce the price of antiretroviral medicines for HIV.

## METHODS

This is a case study based on the medicine atazanavir, which includes the use of a Partnership for Productive Development (PDP). The methodology involved two steps: analysis of the importance of atazanavir for HIV treatment and its share of the MoH budget for ARV, and comparison of the prices paid by the MoH with international reference prices.

Purchases of ARV by the MoH from 2005 to 2013 were analyzed using the records of the General Services Administration Integrated System (SIASG). This system contains information on public purchases by the Federal Public Administration, from the private sector. SIASG does not include purchases from public manufacturers.

The volume and unit prices of annual purchases were used to estimate the total expenditure on ARV and the proportion represented by atazanavir per year. The final volume per year was expressed as number of treatments. The cost of treatment per patient per year (number of capsules per day × 365 × median price) was also calculated, using as reference the 2008 treatment guideline of the Ministry of Health for antiretroviral therapy in adults.

Median prices in Brazilian reais (R$) were adjusted for inflation by the Consumer Price Index (IPCA), using the references provided at the IPEA-data report. The variation rates were calculated for prices and volumes, and the correlation coefficient (t-Student’s test) for the 200 mg capsule was also calculated.^14^

Median prices were converted to the average US dollar rate for the year (IPEA-data) for comparison with international prices. International prices used were as published by Doctors without Borders,^c^ which tracks the lowest prices charged by multinational companies in different countries and by generic alternatives.

The analysis of the PDP for local production of atazanavir aimed to deepen the knowledge about a new Government strategy for price reduction. Among the documents reviewed was the ‘Technical Cooperation Agreement for Sublicensing of Patent Exploitation, Technology Transfer (atazanavir) and Provision signed between the Oswaldo Cruz Foundation and Bristol-Myers Squibb Company’ (hereinafter referred to as the “Agreement”).

The Agreement was obtained through access to information channels and provided by the Brazilian Interdisciplinary Aids Association.

The agreement shows an estimate of a 5.0% reduction in the price of atazanavir per year. This percentage was used to estimate the price reduction from 2012. The analysis enabled inferences about the conditions of the technology transfer to be made.

## RESULTS

In view of total ARV expenditure purchased from the private sector (the majority under a monopoly situation),^d^ the proportion of expenditure on atazanavir ranged from 28.7% in 2008 to 66.5% in 2010, with percentages below 15.0% in 2007 (6.5%), 2009 (13.4%) and 2012 (13.7%) (Table 1).

**Table 1:** Estimates of annual spending of the Ministry of Health with antiretroviral medicines (ARV) acquired in the private sector and the proportion spent with atazanavir. Brazil, 2005 to 2013.

	2005	2006	2007	2008	2009	2010	2011	2012	2013
Total expenses with atazanavir (R$)	245,714,383.80	303,178,200.00	20,109,600.00	112,899,000.00	100,609,380.00	96,522,000.00	128,234,000.00	67,920,375.60	141,095,400.00
Total expenses with ARV (R$)	561,725,242.84	527,067,030.28	310,567,706.67	393,836,424.20	749,426,682.00	145,026,218.52	438,828,079.80	495,598,240.80	490,986,065.79
% of the expenses with atazanavir regarding total expenses with ARV	43.7	57.5	6.5	28.7	13.4	66.5	29.2	13.7	28.7
Other ARV identified on the database	abacavir, amprenavir, didanosina, efavirenz, estavudina, indinavir, lamivudina, nevirapina, ritonavir, tenofovir, zidovudina	abacavir, amprenavir, didanosina, efavirenz, estavudina, lamivudina, nevirapina, ritonavir, tenofovir, tipranavir, saquinavir, zidovudina	abacavir, amprenavir, didanosina, darunavir, efavirenz, estavudina, fosamprenavir, lamivudina, lopinavir/ritonavir, ritonavir, tenofovir, tipranavir, saquinavir, zidovudina	abacavir, amprenavir, darunavir, efavirenz, estavudina, etravirina, fosamprenavir, maraviroque, raltegravir, saquinavir, tenofovir, tipranavir	didanosina, darunavir, etravirina, fosamprenavir, lopinavir/ritonavir, maraviroque, raltegravir, ritonavir, saquinavir, tenofovir, tipranavir	didanosina, efavirenz, estavudina, etravirina, fosamprenavir, lamivudina, maraviroque, ritonavir, saquinavir, tipranavir, zidovudina	didanosina, efavirenz, estavudina, etravirina, fosamprenavir, lopinavir/ritonavir, maraviroque, raltegravir, ritonavir, saquinavir, tipranavir, zidovudina	abacavir, darunavir, didanosina, efavirenz, enfuvirtida, estavudina, etravirina, fosamprenavir, lopinavir, maraviroque, raltegravir, ritonavir, saquinavir, tipranavir, zidovudina	abacavir, darunavir, didanosina, efavirenz, enfrvirtida, etravirina, fosamprenavir, lamivudin, lopinavir, maraviroque, nevirapina, ritonavir, zidovudina

Source: Administration Integrated System of General Services (SIASG). Spending data have not been adjusted by the Consumer Price Index.

The correlation coefficient for the 200 mg capsule, -0.2108 (p = 0.62), suggests no evidence of correlation between rates of change of volume and rates of change in price (Table 2), during the period.

**Table 2:** Estimates of the volume purchased, median price and cost per patient/year of atazanavir 200 mg, 150 mg and 300 mg. Brazil, 2005 to 2013.

Dosage form	2005	2006	2007	2008	2009	2010	2011	2012	2013
Atazanavir 200 mg									
Volume^a^	20,901	18,740	6,041	13,685	2,745	6,575	54,247	5,479	8,219
Rate of volume variation		-0.103	-0.678	1.265	-0.799	1.395	7.250	-0.899	0.500
Median price (R$)^b^	14.13	10.91	6.37	5.41	6.60	4.95	3.89	3.53	3.40
Rate of price variation^c^		-0.228	-0.416	-0.151	0.220	-0.250	-0.214	-0.093	-0.037
Median price (US$)^d^	3.86	3.44	2.34	2.24	2.61	2.36	2.07	1.70	1.58
Cost per patient per year (US$)	2,815.07	2,509.38	1,708.83	1,631.95	1,908.16	1,721.31	2,379.58	1,244.06	1,150.35
Atazanavir 150 mg									
Volume^a^	17,887	38,219	0	22,603	0	0	0	0	0
Rate of volume variation		1.137	-1		-1				
Median price (R$)^b^	13.54	10.45	0	5.75	0	0	0	0	0
Rate of price variation^c^		-0.228	-1		-1				
Median price (US$)^d^	3.70	3.29	0	2.38	0	0	0	0	0
Cost per patient per year (US$)	2,698.15	2,403.70	0	1,735.44	0	0	0	0	0
Atazanavir 300 mg									
Volume^a^	0	0	0	0	24,574	27,945	15,890	27,397	59,260
Rate of volume variation						0.137	-0.431	0.724	1.163
Median price (R$)^b^	0.00	0.00	0.00	0.00	12.71	8.97	6.12	5.78	5.58
Rate of price variation^c^						-0.294	-0.318	-0.056	-0.035
Median price (US$)^d^	0	0	0	0	5.03	4.27	3.26	2.79	2.59
Cost per patient per year (US$)	0	0	0	0	1,836.88	1,557.47	756.15	1,019.91	943.97

^a^ Expressed in estimated number of treatments acquired.

^b^ Values adjusted for inflation through the Consumer Price Index.

^c^ Calculated from the median price in Brazilian reais (R$).

^d^ Calculated by the average dollar of the year.

**Figure 1:**
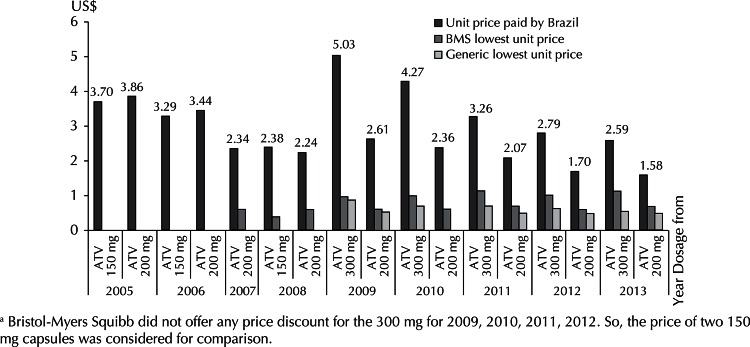
Comparison of the price paid by the Ministry of Health for the atazanavir 150 mg, 200 mg and 300 mga with discount prices offered by Bristol-Myers Squibb and the prices of the generic version. Brazil, 2005 to 2013.

The comparison between prices paid in Brazil, and the lowest price offered by Bristol-Myers Squibb (BMS) (discount price) and the generic version, showed that those paid in Brazil are the highest (Figure 1).

a Bristol-Myers Squibb did not offer any price discount for the 300 mg for 2009, 2010, 2011, 2012. So, the price of two 150 mg capsules was considered for comparison.

**Figure 2:**
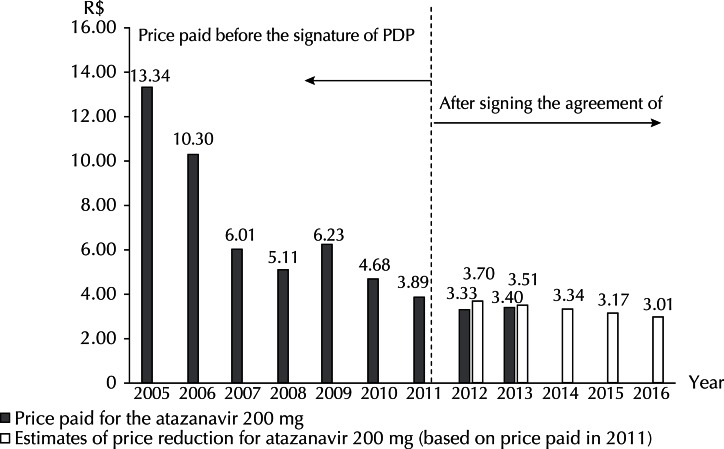
Prices paid for atazanavir 200 mg and reduction estimation after signing the Agreement of Partnership for Productive Development. Brazil, 2005 to 2016.

**Figure 3:**
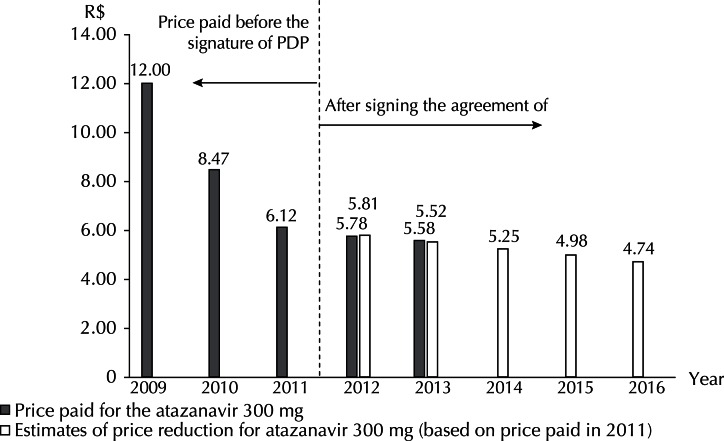
Prices paid for atazanavir 300 mg and reduction estimation after signing the Agreement of Partnership for Productive Development. Brazil, 2009 to 2016.

In relation to the latest strategy adopted in Brazil, price reduction estimates of 5.0% per year and actual prices paid for 2012 and 2013 are presented. The price reductions observed for the 200 mg capsule were greater than the estimated reduction of 5.0% (Figure 2). The amounts paid for the 300 mg capsule in the first two years after the PDP were close to the estimated values (Figure 3).

Although these values indicate consistency with the estimated reduction, nominal values remained virtually constant between 2011 (signing of the PDP), 2012 and 2013 (operation of PDP). The prices paid in the three years for the 200 mg capsule were R$3.47, R$3.34 and R$3.40, respectively.

The prices paid for the 300 mg capsule in the three years were R$5.46, R$5.46 and R$5.58, respectively. The volume purchased in 2013 increased by more than 31,863 treatments from 2012 (Table 2).

## DISCUSSION

Atazanavir is a protease inhibitor, used with ritonavir as a booster. Atazanavir has been recommended by the World Health Organization (WHO) for the treatment of HIV since 2006 (Treatment Guidelines for adults and adolescents), included among the second-line regimens. It was included in the WHO Essential Medicines List in 2009 for adults and children, respectively, in the 16^th^ and 2^nd^ editions of the Model List.

In Brazil, atazanavir was incorporated in the treatment guidelines of 2003, indicated as one of the options for first-line or as part of second-line treatment. Its adoption in the country was almost simultaneous with the approval of the medicine by the FDA (United States Food and Drug Administration), in June 2003. Between the arrival of the first batches of atazanavir in January 2004 and December 2006, the number of people who used the medicine increased from 6,000 in July 2004 to 25,000 people in 2006.^e^

The data in Table 1 reflects an estimate of the number of treatments purchased, rather than used, in that year. However, considering that the MoH provides more than 20 ARV, the expenditure on atazanavir is significant.

The BMS holds the exclusive right to market atazanavir in Brazil because it is the patent holder of the medicine (effective until 2016), while the MoH is the sole buyer.

The use of purchase volumes should be considered as a strategy for price reductions. It would be expected that the larger the purchase volumes, the lower the price paid.^12^ Based on this assumption, in 1996, the MoH chose to centralize ARV purchases as a strategy to reduce prices and ensure availability.^17^

However, the correlation coefficient of variation rates, of purchase volumes and prices of 200 mg atazanavir, suggests lack of evidence of correlation (Table 2). Therefore, the monopoly power conferred by the patent undermined the purchasing power of the MoH.

The prices of ARV purchased in Brazil between 1998 and 2001^9,11^ were sensitive to the volume of purchase. As patented medicines were being incorporated into the MoH treatment guidelines, price reductions became smaller. This indicates the purchasing power of the government as the sole buyer of large volumes of ARV is effective on price reductions when there is competition.

When a new patented medicine is incorporated in the treatment guidelines, the Government is required by law to ensure universal access to it. The company is in an advantageous position, because the Government will have to buy the medicine, even if prices are high. This indicates inelasticity of demand to price.

The existence of information on prices and the availability of generic versions in the national and international market are other elements of a price-reduction strategy.^21^ The first allows the government to oppose the price offered by the company; the second enables the government to acquire cheaper alternatives by the use of compulsory license, even if no immediate local production capacity exists.

Atazanavir prices paid by Brazil are higher than the lowest price offered by BMS (discount price) and the generic version (Figure 1). However, Brazil is excluded from the possibility of acquiring either of these alternatives, which weakens its bargaining power in the negotiation. The exclusion is determined by the fact that Brazil is not among the countries benefitting from the BMS price discrimination policy, and because the companies producing the generic versions are under voluntary license agreements with BMS. These licenses restrict the geographical scope of marketing to African countries and to India.^f^

In this scenario, the challenge is to overcome the patent barrier. There are options to overcome the patents on medicines^g,h,i^ and press for price reductions.^5^ One of them is the use of a pre-grant opposition (in Brazil, so-called “support to examination”) or a post-grant opposition (“nullity”) to the granting of the patent. Another option is the issue of compulsory license that, in Brazil, is generally subject to the availability of a generic version in the international market that can meet the country’s need, until local production ensures the supply. This is because the local pharmaceutical industry would need a period to start production.

Alternatively, the earlier use of a TRIPS flexibility, known as the Bolar exception,^2^ allows for product development during the patent term, facilitating the issuance of a compulsory license and the negotiation process. It would make it possible to produce the medicine more quickly and locally, in addition to furnishing better estimates of production cost. These options do not seem to have been considered among the strategies to increase the Government’s bargaining power for atazanavir price reductions.

An alternative used by the Brazilian Government, since 2008, when the “Productive Development Policy” was issued and, later, the “Greater Brazil Plan” in 2011, has been to stimulate local production of priority medicines adopted by SUS^5,23^ through PDP.

The adoption of PDP for medicines includes both products with and without a monopoly situation. The transfer of the technology must involve a technology holder, a national private producer for production of the active pharmaceutical ingredient and a public producer for the final product, which, in turn, can market the product exclusively in the public sector. In summary, the Government’s purchasing power was used to induce local production for medicines and active pharmaceutical ingredients because it guaranteed a market for the preferred producer, for products defined as priorities adopted by MoH (MoH Ordinances 978/2008, 1,284/2010 and subsequently 3,089/2013).

Promoting local production and reducing dependence on the external pharmaceutical industry have been the goals of PDP in Brazil. However, the monopoly created by the market guarantee to a preferred producer might have a negative impact on prices.^11,16^ It creates a monopoly for non-patented products and maintains and strengthens the monopoly for those that are patented.

Some PDP have been announced since 2009. However, the specific objectives for their use were only initially defined in 2012 (MoH Ordinance 837/2012). The objectives included: the streamlining of the purchasing power of the Government; greater cooperation on technology development and exchanges of knowledge among private and public producers; local production of strategic medicines, which are also expensive and crucial to ensure availability; and progressive negotiations for significant price reductions.

The way in which technology development cooperation and exchange of knowledge would be were not detailed, among the specific objectives. The incorporation of technology in an isolated manner is not enough to increase the government’s bargaining power. If there is no forecast of additional investments in technological training and workforce training, the transfer of technology in fact will not occur.^8^ Without the guarantee of technological and capacity accumulation, local companies will not be empowered to collaborate in the development of newer and better technologies.^15,j^

In addition, only technological accumulation will help to improve the information asymmetry in technology transfer agreements between technology buyer and seller.^j^ It will also allow the estimate of medicines production costs based on knowledge of companies margins and increase the potential to issue compulsory licenses through local production, if negotiations are not advantageous.

The Brazilian Government announced its intention to implement 10 PDP for local production of ARV between 2009 and the end of 2012.^k^ Among these, is the atazanavir PDP, signed in 2011 between BMS and Farmanguinhos – Instituto de Tecnologia em Fármacos of the Fundação Oswaldo Cruz (public manufacturer), for the voluntary license of the existing patent and technology transfer of the active pharmaceutical ingredient and the 200 mg and 300 mg capsule dosage forms.^l^ The present study focuses on aspects of that Agreement related to prices and possible strengthening of the underlying monopoly.

The Agreement establishes among its results, a 5.0% per year reduction over five years for both dosage forms. Assuming that the Agreement entered into force in January 2012 and that the starting price was the one paid in 2011, registered in SIASG, it was possible to estimate, by the variation in unit prices, a 25.0% reduction until 2016 for the two dosage forms (Figures 2 and 3). Although the rates for 2012 and 2013 indicate a consistency with the estimated reduction when adjusted by the Consumer Price Index, in nominal values, prices are almost constant for the medicines between 2011 and 2013. In practice, the price reduction is more of reflection of the adjustments to the inflation rate.

The most significant price reductions were achieved in the period prior to the signing of PDP. The reduction for the 200 mg capsule was 37.6% between 2009 and 2011. For the 300 mg capsule, the reduction was 49.0% for the same period.

Previous studies on voluntary licensing of ARV^m,n^ have highlighted that the technology holder negotiates restrictive clauses, which may limit the possibility of further price reduction. The agreement between Farmanguinhos and BMS explicitly prevents the production of other dosage forms or fixed-dose combinations, than the 200 mg and 300 mg capsules. However, a fixed-dose combination of atazanavir and ritonavir in heat-stable tablets was included in the WHO treatment guidelines of 2013. If this combination becomes the preferred option and is adopted in the MoH guideline, the capsules produced by Farmanguinhos will soon be obsolete.

Some terms of the Agreement may limit the possibility of further price reduction. It is mandatory for Farmanguinhos to buy 100% of the MoH demand in the first three years after the marketing approval from BMS. In the fourth and fifth years, BMS will continue to supply 50.0% of the MoH’s demand. If there were any delay in the process of technology transfer to Farmanguinhos or in obtaining the marketing approval, BMS would be ensured the market, even after the patent expiration. The patent will expire soon, therefore, the voluntary license represented an opportunity for BMS to exploit the remaining commercial value of the patent.

At the end of 2013, the MPP has negotiated a voluntary license for atazanavir with BMS containing less restrictive provisions than those provided for in the Agreement with Farmanguinhos, as it establishes the possibility of the licensee to produce any kind of dosage form and fixed doses combinations.^o^

Some issues deserve further analysis. One of them is whether a PDP is the most appropriate strategy to overcome the patent barriers and to achieve price reductions. The Government, by being bound to an agreement, gave up the possibility of adopting other antimonopoly strategies, which may be called for if the national and international environment becomes more competitive.

The second is the interface between local production and access. A review of the international literature^p^ explored this relationship and shows that for some cases the benefit of local production in relation to cost savings is doubtful in the short term, providing examples including Brazil. One the one side, it can be argued that local production can ensure availability of the medicine in the national market. In practice, this availability already exists, because atazanavir, as well as other priority products for PDP, is imported and the public market is attractive enough for multinational companies to continue to supply. From another side, local production could improve the bargaining power in the public purchase of medicines under a monopoly situation, in addition to representing a possibility of strategic supply in the presence of importation difficulties.

The volume of acquisition and centralized purchases of patented ARV seem to have little or no effect on price reduction. The case study shows the complexity and the difficulties faced by health authorities to reduce the price of priority medicines in a limited-competition environment. A multi-pronged approach is required to achieve price reductions. The use of TRIPS flexibilities to overcome patent barriers, such as patent oppositions, Bolar exception and compulsory licenses, which were not considered in this case, should also be used as instruments for strengthening the MoH bargaining power in price negotiations.

The Agreement, which establishes the PDP for atazanavir, raises several questions regarding the use of these partnerships solely to reduce prices and overcome patent barriers. The terms on which Brazil enters into a PDP can inhibit companies from adhering to international voluntary licenses held by MPP. Further research is needed to understand how these clauses were negotiated and which strategies could have been exploited to expand the ability of the Government to obtain more favorable clauses from the technology holder.

If the Government pursues the option of strengthening the local production of medicines through the PDP’ strategy, it should also consider research and development investments that stimulate technological accumulation. This would strengthen the Government’s bargaining power to negotiate medicines prices under a monopoly situation and would help the sustainability of the SUS universal access policy.

## Footnotes

a Santos RSL. Sustentabilidade do Programa Nacional de DST/aids: análise da capacidade de oferta e preços dos medicamentos antirretrovirais [dissertation]. Rio de Janeiro (RJ): Instituto de Economia da Universidade Federal do Rio de Janeiro; 2010.

b Medicines Patent Pool [Internet]. About the MPP. [cited 2014 Feb 23]. Available from: http://www.medicinespatentpool.org/about

c Médecins San Frontières, Access Campaign. Untangling the web of antirretroviral price reductions [internet]. 17.ed. Geneva: MSF; 2014 [cited 2014 Nov 15]. Available from: https://www.msfaccess.org/sites/default/files/MSF_UTW_17th_Edition_4_b.pdf

d Villardi P. Panorama do status patentário e registro sanitário dos medicamentos antirretrovirais no Brasil: implicações para o acesso e a política industrial no Brasil. Rio de Janeiro (RJ): Associação Brasileira Interdisciplinar de Aids; 2012 [cited 2014 Jun 12]. Available from: http://www.deolhonaspatentes.org.br/media/file/Publica%C3%A7%C3%B5es/Publica%C3%A7%C3%A3o%20Pedro_Final_23OUT.pdf

e Scheffer MC. Aids, tecnologia e acesso sustentável a medicamentos: a incorporação dos antirretrovirais no Sistema Único de Saúde [thesis]. São Paulo (SP): Faculdade de Medicina da Universidade de São Paulo; 2008.

f Médecins Sans Frontières, Access Campaign. Untangling the web of antiretroviral price reductions. 13.ed. Geneva; 2010 [cited 2013 Apr 25]. Available from: http://d2pd3b5abq75bb.cloudfront.net/2012/07/16/14/39/31/171/UTW_13_ENG_Jul2010.pdf

g World Health Organization. How to develop and implement a national drug policy: updates and replaces: guidelines for developing national drug policies, 1988. 2.ed. Geneva; 2013 [cited 2014 Jan 15]. Available from: http://apps.who.int/medicinedocs/pdf/s2283e/s2283e.pdf

h Joint United Nations Programme on HIV/AIDS. Doha+10 trips flexibilities and access to antiretroviral therapy: lessons from the past, opportunities for the future. Geneva: Unaids; 2011 [cited 2014 Jan 20]. Available from: http://www.unaids.org/en/media/unaids/contentassets/documents/unaidspublication/2011/JC2260_DOHA+10TRIPS_en.pdf

i United Nations Conference on Trade and Development (UNCTAD). Using intellectual property rights to stimulate pharmaceutical production in developing countries: a reference guide. Geneva: United Nations; 2011 [cited 2014 Jan 20]. Available from: http://unctad.org/en/Docs/diaepcb2009d19_en.pdf

j Foray D. Technology transfer in the TRIPS age: the need for new types of partnerships between the least developed and most advanced economies. Geneva: ICTSD; 2009 [cited 2014 Jan 20]. (ICTSD Intellectual Property and Sustainable Development Series; issue paper nº 23). Available from: http://ictsd.org/downloads/2012/02/technology-transfer-in-the-trips-age.pdf

k Ministério da Saúde. Propostas de Projetos de Parcerias para o Desenvolvimento Produtivo (PDP) aprovadas de 2009 a 2014. Brasília (DF); 2015. [cited 2015 Oct 26]. Available from: http://portalsaude.saude.gov.br/images/pdf/2015/outubro/01/Propostas-de-projeto-de-PDP-aprovadas-de-2009-a-2014-01-10-2015.pdf

l Agência Fiocruz de Notícias. Fundação assina acordo para produção do medicamento Atazanavir. Rio de Janeiro (RJ): Fiocruz; 2011 [cited 2013 Mar 26]. Available from: http://www.agencia.fiocruz.br/funda%C3%A7%C3%A3o-assina-acordo-para-produ%C3%A7%C3%A3o-do-medicamento-atazanavir

m Park C, Moon S, Burrone E, Boulet P, Juneja S, ‘t Hoen E et al. Voluntary licensing: an analysis of current practices and key provisions in antiretroviral voluntary licences. Geneva: Medicines Patent Pool; 2012 [cited 2013 Oct 27]. Available from: http://www.medicinespatentpool.org/wp-content/uploads/Current-Practice-and-Key-Provisions-in-ARV-VLs.pdf

n Médecins Sans Frontières, Access Campaign. Barriers to accessing generics: the restrictions posed by certain voluntary license agreements. Geneva; 2012 [cited 2015 Oct 26]. Available from: http://www.msfaccess.org/sites/default/files/MSF_assets/HIV_AIDS/Docs/AIDS_report_UTW15_ENG_2012.pdf

o Medicines Patent Pool. License and technology transfer agreement. Geneva; 2013 [cited 2014 Jan 10]. Available from: http://www.medicinespatentpool.org/wp-content/uploads/MPP-License-and-technology-transfer-agreement-Signed.pdf

p World Health Organization. Local production for access to medical products: developing a framework to improve public health. Geneva; 2011 [cited 2014 Jan 10]. Available from: http://www.who.int/phi/publications/Local_Production_Policy_Framework.pdf
