# ERRATUM

**DOI:** 10.1590/S01518-8787.2016050005459err

**Published:** 2016-04-15

In the article: “**Strategies for price reduction of HIV medicines under a monopoly situation in Brazil**”, volume 49 (2015), in the Table 2 (Port/Engl) and in the Figures 2 and 3 (English).

Table 2. In the row 8, column 8, where it reads 2.379,58, it should read 1.512,30.

Table 2. In the row 22, column 8, where it reads 756,15, it should read 1.189,79

Figures 2 and 3 (English version), where it reads “After signing the agreement of”, it should read “After signing the PDP Agreement”.

