# Supplementary material for: Strategies for price reduction of HIV medicines under a monopoly situation in Brazil
Source: Rev Saude Publica. 2016 Mar 1;49:86. doi: 10.1590/S0034-8910.2015049005459 (PMC4687827; doi:10.1590/S0034-8910.2015049005459)
Supplement: Translation

# Translation: Estratégias de redução de preços de medicamentos para aids em situação de monopólio no Brasil

**Authors:** Gabriela Costa Chaves, Lia Hasenclever, Claudia Garcia Serpa Osorio-de-Castro, Maria Auxiliadora Oliveira

**Affiliations:** I Departamento de Política de Medicamento e Assistência Farmacêutica. Escola Nacional de Saúde Pública Sergio Arouca. Fundação Oswaldo Cruz. Rio de Janeiro, RJ, Brasil; II Grupo de Economia da Inovação. Instituto de Economia. Universidade Federal do Rio de Janeiro. Rio de Janeiro, RJ, Brasil

**Keywords:** Fármacos Anti-HIV, provisão & distribuição, Preço de Medicamento, Custos de Cuidados de Saúde, Propriedade Intelectual, Política Nacional de Medicamentos

## Abstract

**OBJETIVO:**

Analisar as estratégias governamentais para redução de preço de medicamentos antirretrovirais para aids no Brasil.

**MÉTODOS:**

Realizada análise das compras de medicamentos antirretrovirais pelo Ministério da Saúde, de 2005 a 2013. Foram analisados o gasto e o custo do tratamento por ano e comparados com os preços internacionais para o atazanavir. Foram estimadas as reduções com base no contrato da Parceria para Desenvolvimento Produtivo para obtenção de licença voluntária de patente e transferência de tecnologia do atazanavir.

**RESULTADOS:**

O atazanavir teve peso expressivo nos gastos com antirretrovirais adquiridos no setor privado. Os preços praticados no Brasil foram mais altos que aqueles de referência internacional e não houve evidências da relação entre volume de compra e preço pago pelo Ministério da Saúde, por ser medicamento patenteado. Em relação à estratégia mais recente para reduzir preços, envolvendo produção local da cápsula de 200 mg, as reduções foram menores do que as estimadas. Quanto à cápsula de 300 mg, os valores pagos nos dois primeiros anos após a Parceria para Desenvolvimento Produtivo foram próximos aos estimados. Os preços em valores nominais mantiveram-se praticamente constantes entre 2011 (assinatura da Parceria para Desenvolvimento Produtivo), 2012 e 2013 (após estabelecida a Parceria).

**CONCLUSÕES:**

A redução do preço de medicamentos é complexa em ambiente de concorrência limitada. O uso da Parceria para Desenvolvimento Produtivo como método para aumentar a capacidade de produção local e reduzir preços levanta questões em relação à redução efetiva dos preços e ao enfrentamento da barreira patentária. Investimentos em pesquisa e desenvolvimento que possam estimular a acumulação tecnológica devem ser considerados pelo governo para fortalecer seu poder de barganha ao negociar preços de medicamentos em situação de monopólio.

## INTRODUÇÃO

O Brasil garante acesso universal e gratuito aos medicamentos antirretrovirais (ARV) e outros necessários ao tratamento e controle da infecção pelo HIV no Sistema Único de Saúde (SUS) desde 1996.^7^ Ao longo dos anos, preços, custos e gastos crescentes com medicamentos em situação de monopólio ameaçam a sustentabilidade financeira da resposta à epidemia no País.^7,18,20^

Os gastos do Ministério da Saúde (MS) têm aumentado devido ao aumento do número de pessoas vivendo com HIV em terapia ARV, à emergência de cepas virais resistentes aos esquemas de primeira e segunda linha e à incorporação de novos ARV.^6,11^ A necessidade de migrar para esquemas terapêuticos de segunda e terceira linhas, em função da resistência viral, significa passar para opções envolvendo ARV mais caros, geralmente importados e em situação de monopólio.^7^

No cenário atual de vigência plena do Acordo sobre os Aspectos dos Direitos de Propriedade Intelectual Relacionados ao Comércio (Acordo TRIPS), da Organização Mundial do Comércio (OMC), os ARV mais novos estarão em situação de monopólio no Brasil e internacionalmente. Isso aumenta a dificuldade de negociar e obter reduções de preços.^3,11,18^

Medicamentos ARV em situação de monopólio são aqueles ofertados por um único fornecedor, em geral, por estarem sujeitos à proteção patentária (pedido de patente depositado ou patente concedida no País).

Estudos analisaram a evolução dos gastos do MS com ARV e os determinantes dos preços em séries históricas de 1996 a 2009.^6,9-11,16,a^ Os ARV em situação de monopólio tiveram seus preços reduzidos inicialmente, mas esse ganho foi perdido ao longo do tempo. Foi observado aumento expressivo do gasto com ARV patenteados, que chegou a comprometer 80,0% dos recursos do MS destinados à compra de ARV em 2004 e 2005.^16^

Os medicamentos que possuem múltiplos fornecedores são mais sensíveis a variáveis como volume de compra. Já o preço dos medicamentos em situação de monopólio é pouco sensível ao volume de compra, mas reage quando o poder de barganha é fortalecido pela apresentação de evidência sobre os custos de produção, ameaça e emissão de licença compulsória, entre outros, em negociações de preços.^16^ O poder de barganha se reduz quando a capacitação tecnológica e industrial local ou as alternativas de fornecedores não existem ou são restritas.^9,11,16^

Iniciativas internacionais para enfrentar os preços dos ARV em situação de monopólio foram implementadas em países em desenvolvimento nos anos 2000. Grandes doadores, como o Fundo Global de Combate ao HIV, tuberculose e malária e a Central Internacional de Compras de Medicamentos (Unitaid), tiveram papel relevante na dinâmica dos preços, mediante aquisição de grandes quantidades e garantia de mercado para estimular o desenvolvimento de combinações em doses fixas e versões pediátricas.^25^

As empresas farmacêuticas multinacionais, por sua vez, adotaram políticas de discriminação de preços (“preços diferenciados”), criando categorias de descontos segundo parâmetros por elas estabelecidos, que consideram o nível de desenvolvimento do país e a prevalência do HIV.^13,24^ No entanto, os critérios não são seguidos por todas as empresas pois alguns países, que são elegíveis para uma empresa, podem não ser para outras.

Iniciativa mais recente para superar a barreira patentária e incentivar a disponibilidade de combinações em doses fixas e formas pediátricas foi a criação do *Medicines Patent Pool* (MPP), que visa negociar licenças voluntárias com empresas farmacêuticas multinacionais para promover a concorrência genérica e reduzir preços.^b^

Embora essas iniciativas internacionais apresentem resultados na ampliação do acesso aos ARV e no enfrentamento dos preços altos, o Brasil é excluído de todas elas e teve que buscar suas próprias estratégias para enfrentar essas barreiras.

As principais estratégias do governo brasileiro para redução de preços dos ARV em situação de monopólio, de 2001 a 2007, incluíram: negociação de preços com as empresas farmacêuticas multinacionais com ameaça de emissão de licença compulsória, substanciada em estimativas do custo de produção^19^ e preços de referência internacionais; contestação de pedido de patente por laboratório público oficial;^1^ e emissão de licença compulsória para importação e posterior produção local do medicamento.^11,22^

O estudo das estratégias governamentais para assegurar o acesso a ARV em um país como o Brasil abrange a compreensão do processo de incorporação da tecnologia no sistema de saúde, das abordagens para garantir a disponibilidade (em contexto de manutenção e expansão do tratamento) e das iniciativas para enfrentamento dos preços dos produtos em situação de monopólio, incluindo esforços para a produção local.

O objetivo deste estudo foi analisar as estratégias governamentais para redução de preço de medicamentos antirretrovirais para aids.

## MÉTODOS

Utilizou-se o caso do medicamento atazanavir, que inclui uma Parceria para Desenvolvimento Produtivo (PDP). A metodologia envolveu duas etapas: analisar a importância do atazanavir para o tratamento da aids e seu peso no orçamento, e comparar os preços pagos pelo MS com outros de referência internacional.

As compras dos ARV realizadas pelo MS de 2005 a 2013 foram analisadas por meio dos registros do Sistema Integrado de Administração de Serviços Gerais (SIASG). Este sistema contém informações de compras públicas da Administração Pública Federal, efetuadas no setor privado. O SIASG não inclui compras por convênios com laboratórios públicos oficiais.

O volume e os preços unitários de compra anual foram utilizados para estimar o gasto total com ARV e a proporção do gasto com o atazanavir por ano. O volume final por ano foi expresso em número de tratamentos adquiridos. O custo do tratamento por paciente por ano (número de cápsulas por dia × 365 × preço mediano) também foi calculado, adotando-se como referência o protocolo oficial do Ministério da Saúde, de 2008, para terapia antirretroviral em adultos.

Os preços medianos em reais (R$) foram ajustados à inflação pelo Índice de Preços ao Consumidor Amplo (IPCA) do período para comparação entre os anos, utilizando as referências disponibilizadas no IPEA-data. Foram calculadas as taxas de variação para os preços e os volumes, assim como o coeficiente de correlação (teste t-Student) para a cápsula de 200 mg.^14^

Os preços medianos foram convertidos para o dólar médio do ano (IPEA-data) para comparação com os preços internacionais. Adotou-se como referência de preços internacionais aqueles publicados pelos Médicos Sem Fronteiras,^c^ que monitora os menores preços praticados pelas empresas multinacionais de diferentes países e com alternativas genéricas.

A análise da PDP para produção local do atazanavir visou aprofundar o conhecimento da nova estratégia governamental para redução de preços, a partir do estudo de caso. Entre os documentos analisados, destaca-se o Acordo de Cooperação Técnica para Sublicenciamento de Exploração de Patente, Transferência de Tecnologia (do atazanavir) e Fornecimento que entre si celebram a Fundação Oswaldo Cruz e a Bristol-Meyers Squibb Company (doravante designado como “Acordo”).

O Acordo foi obtido por meio de canais de acesso à informação e cedido pela equipe da Associação Brasileira Interdisciplinar de aids.

No Acordo, consta a estimativa de redução de 5,0% do preço do atazanavir por ano de sua vigência. Esse percentual foi utilizado para estimar a redução do preço a partir de 2012. Sua análise possibilitou inferir informações sobre as condições da transferência de tecnologia.

## RESULTADOS

A proporção do gasto do atazanavir em relação aos gastos com ARV adquiridos pelo setor privado (maioria em situação de monopólio)^d^ oscilou entre 28,7% em 2008 e 66,5% em 2010, com percentuais abaixo de 15,0% em 2007 (6,5%), 2009 (13,4%) e 2012 (13,7%) (Tabela 1).

**Tabela 1:** Estimativa do gasto anual do Ministério da Saúde com antirretrovirais (ARV) adquiridos no setor privado e proporção do gasto com atazanavir. Brasil, 2005 a 2013.

	2005	2006	2007	2008	2009	2010	2011	2012	2013
Gasto total com atazanavir (R$)	245.714.383,80	303.178.200,00	20.109.600,00	112.899.000,00	100.609.380,00	96.522.000,00	128.234.000,00	67.920.375,60	141.095.400,00
Gasto total com ARV (R$)	561.725.242,84	527.067.030,28	310.567.706,67	393.836.424,20	749.426.682,00	145.026.218,52	438.828.079,80	495.598.240,80	490.986.065,79
% do gasto com atazanavir em relação ao gasto total com ARV	43,7	57,5	6,5	28,7	13,4	66,5	29,2	13,7	28,7
Demais ARV identificados na base	abacavir, amprenavir, didanosina, efavirenz, estavudina, indinavir, lamivudina, nevirapina, ritonavir, tenofovir, zidovudina	abacavir, amprenavir, didanosina, efavirenz, estavudina, lamivudina, nevirapina, ritonavir, tenofovir, tipranavir, saquinavir, zidovudina	abacavir, amprenavir, didanosina, darunavir, efavirenz, estavudina, fosamprenavir, lamivudina, lopinavir/ritonavir, ritonavir, tenofovir, tipranavir, saquinavir, zidovudina	abacavir, amprenavir, darunavir, efavirenz, estavudina, etravirina, fosamprenavir, maraviroque, raltegravir, saquinavir, tenofovir, tipranavir	didanosina, darunavir, etravirina, fosamprenavir, lopinavir/ritonavir, maraviroque, raltegravir, ritonavir, saquinavir, tenofovir, tipranavir	didanosina, efavirenz, estavudina, etravirina, fosamprenavir, lamivudina, maraviroque, ritonavir, saquinavir, tipranavir, zidovudina	didanosina, efavirenz, estavudina, etravirina, fosamprenavir, lopinavir/ritonavir, maraviroque, raltegravir, ritonavir, saquinavir, tipranavir, zidovudina	abacavir, darunavir, didanosina, efavirenz, enfuvirtida, estavudina, etravirina, fosamprenavir, lopinavir, maraviroque, raltegravir, ritonavir, saquinavir, tipranavir, zidovudina	abacavir, darunavir, didanosina, efavirenz, enfrvirtida, etravirina, fosamprenavir, lamivudin, lopinavir, maraviroque, nevirapina, ritonavir, zidovudina

Fonte: Sistema Integrado de Administração de Serviços Gerais. Dados do gasto não foram ajustados pelo Índice de Preços ao Consumidor Amplo.

O cálculo do coeficiente de correlação para a apresentação de 200 mg de -0,2108 (p = 0,62) sugere não haver evidências da existência de correlação entre taxas de variação do volume adquirido no período e as taxas de variação do preço (Tabela 2).

**Tabela 2:** Estimativa do volume adquirido, mediana do preço e custo por paciente/ano do atazanavir 200 mg, 150 mg e 300 mg. Brasil, 2005 a 2013.

Apresentação	2005	2006	2007	2008	2009	2010	2011	2012	2013
Atazanavir 200 mg									
Volume^a^	20.901	18.740	6.041	13.685	2.745	6.575	54.247	5.479	8.219
Taxa de variação do volume		-0,103	-0,678	1,265	-0,799	1,395	7,250	-0,899	0,500
Mediana do preço (R$)^b^	14,13	10,91	6,37	5,41	6,60	4,95	3,89	3,53	3,40
Taxa de variação do preço^c^		-0,228	-0,416	-0,151	0,220	-0,250	-0,214	-0,093	-0,037
Mediana do preço (US$)^d^	3,86	3,44	2,34	2,24	2,61	2,36	2,07	1,70	1,58
Custo por paciente por ano (US$)	2.815,07	2.509,38	1.708,83	1.631,95	1.908,16	1.721,31	2.379,58	1.244,06	1.150,35
Atazanavir 150 mg									
Volume^a^	17.887	38.219	0	22.603	0	0	0	0	0
Taxa de variação do volume		1,137	-1		-1				
Mediana do preço (R$)^b^	13,54	10,45	0	5,75	0	0	0	0	0
Taxa de variação do preço^c^		-0,228	-1		-1				
Mediana do preço (US$)^d^	3,70	3,29	0	2,38	0	0	0	0	0
Custo por paciente por ano (US$)	2.698,15	2.403,70	0	1.735,44	0	0	0	0	0
Atazanavir 300 mg									
Volume^a^	0	0	0	0	24.574	27.945	15.890	27.397	59.260
Taxa de variação do volume						0,137	-0,431	0,724	1,163
Mediana do preço (R$)^b^	0,00	0,00	0,00	0,00	12,71	8,97	6,12	5,78	5,58
Taxa de variação do preço^c^						-0,294	-0,318	-0,056	-0,035
Mediana do preço (US$)^d^	0	0	0	0	5,03	4,27	3,26	2,79	2,59
Custo por paciente por ano (US$)	0	0	0	0	1.836,88	1.557,47	756,15	1.019,91	943,97

^a^ Expresso em estimativa do número de tratamentos adquiridos.

^b^ Valores corrigidos pelo Índice de Preços ao Consumidor Amplo.

^c^ Calculada a partir da mediana do preço em reais (R$).

^d^ Calculado pelo dólar médio do ano.

A comparação entre os preços pagos no Brasil em relação ao menor preço ofertado pela Bristol-Myers Squibb (BMS) (preço de desconto) e o da versão genérica mostrou que aqueles praticados no País são os mais altos (Figura 1).

**Figura 1:** Comparação do preço pago pelo Ministério da Saúde para o atazanavir 150 mg, 200 mg e 300 mga com os preços de desconto ofertado pela Bristol-Myers Squibb e os preços da versão genérica. Brasil, 2005 a 2013.

a Não havia preço de desconto oferecido pela Bristol-Myers Squibb para a apresentação de 300 mg para 2009, 2010, 2011, 2012. Por isso, foram considerados o preço de duas cápsulas de 150 mg para comparação.

Em relação à estratégia mais recente adotada no Brasil, foram apresentadas as estimativas de redução de 5,0% ao ano e os preços pagos em 2012 e 2013. As reduções observadas para a cápsula de 200 mg foram menores do que a redução estimada de 5,0% (Figura 2). Os valores pagos para a cápsula de 300 mg nos dois primeiros anos após a PDP foram próximos aos valores estimados (Figura 3).

**Figura 2:** Preços pagos pelo atazanavir 200 mg e estimativa de redução após assinatura do acordo da Parceria para Desenvolvimento Produtivo. Brasil, 2005 a 2016.

**Figura 3:** Preços pagos pelo atazanavir 300 mg e estimativa de redução após assinatura do acordo da Parceria para Desenvolvimento Produtivo. Brasil, 2009 a 2016.

Embora esses valores apontem consistência com os estimados de redução, valores nominais mantiveram-se praticamente constantes entre 2011 (assinatura da PDP), 2012 e 2013 (após PDP). Os preços pagos nos três anos para a cápsula de 200 mg foram R$3,47, R$3,34 e R$3,40, respectivamente.

Os preços pagos para a cápsula de 300 mg nos três anos foram R$5,46, R$5,46 e R$5,58, respectivamente. O volume comprado em 2013 foi de 31.863 tratamentos a mais do que em 2012 (Tabela 2).

## DISCUSSÃO

O atazanavir pertence à classe dos inibidores de protease, utilizado com o ritonavir como potencializador (*booster* ). O atazanavir foi recomendado pela Organização Mundial da Saúde (OMS), no protocolo para adultos e adolescentes, para o tratamento do HIV desde 2006, incluído entre os esquemas de segunda linha. A inclusão na Lista de Medicamentos Essenciais ocorreu em 2009 para adultos e crianças, respectivamente, nas 16ª e 2ª edições da Lista Modelo.

No Brasil, ele foi incorporado no consenso terapêutico de 2003, indicado como uma das opções para primeira linha ou como parte do esquema de segunda linha. Sua adoção no País foi quase simultânea à aprovação do medicamento pela FDA (autoridade regulatória dos Estados Unidos) em junho de 2003. Entre a chegada do primeiro lote de atazanavir em janeiro de 2004 e dezembro de 2006, o número de pessoas que usava esse medicamento passou de 6.000 em julho de 2004 para 25.000 pessoas em 2006.^e^

Os dados da Tabela 1 refletem a dinâmica do processo de compras e não uso do medicamento. No entanto, o gasto com o atazanavir é expressivo, considerando que o elenco do MS é composto por mais de 20 ARV.

A BMS tem direito de exclusividade sobre a comercialização do atazanavir no Brasil porque é titular da patente principal do medicamento (vigência até 2016), sendo o MS o único comprador no Brasil.

O volume da compra deve ser considerado como estratégia de redução de preços. Espera-se que, quanto maior o volume da compra, menor o preço a ser pago.^12^ Com base nesse pressuposto, em 1996 o MS optou por centralizar as compras de ARV como uma estratégia para reduzir preços e garantir a disponibilidade.^17^

Entretanto, o coeficiente de correlação das taxas de variação dos volumes da compra e dos preços do atazanavir 200 mg sugere falta de evidências da correlação (Tabela 2). Portanto, o poder de monopólio conferido pela patente neutralizou o poder de compra do MS.

Os preços dos ARV adquiridos no Brasil entre 1998 e 2001^9,11^ foram sensíveis ao volume de compra. Conforme os medicamentos patenteados foram sendo incorporados ao elenco do MS, a redução de preço tornou-se menor. O poder de compra do governo como único comprador de grande volume de ARV só tem efeito sobre o preço quando existe concorrência.

Quando um novo medicamento é inserido no consenso terapêutico e é patenteado, o governo é obrigado por lei a garantir o acesso a ele. A empresa tem posição vantajosa, pois sabe que o governo será obrigado a comprar o medicamento, ainda que os preços sejam altos. Isso caracteriza inelasticidade da demanda ao preço.

A existência de informação sobre preços e a disponibilidade de versões genéricas no mercado nacional e internacional são outros elementos de uma estratégia para redução de preços.^21^ A primeira permite ao governo confrontar o preço oferecido pela empresa; a segunda habilita o governo a adquirir alternativas mais baratas mediante uso da licença compulsória, mesmo que não haja capacidade local imediata de produção.

Os preços do atazanavir pagos pelo Brasil são mais altos do que o menor preço ofertado pela BMS (preço de desconto) e da versão genérica (Figura 1). No entanto, o Brasil está excluído da possibilidade de adquirir uma dessas alternativas, o que enfraquece seu poder de barganha na negociação. A exclusão é determinada pelo fato de o Brasil não estar entre os países beneficiados pela política de discriminação de preços da BMS e porque as empresas produtoras da versão genérica são licenciadas por ela. Essas licenças restringem o escopo geográfico de comercialização a países da África e Índia.^f^

Neste cenário, o desafio é superar a barreira patentária. Quando um medicamento é patenteado, existem opções para superar essa barreira^g,h,i^ e exercer pressões sobre o preço.^5^ Uma delas é a contestação prévia (subsídio ao exame) ou posterior (nulidade) à concessão da patente. Outra é a emissão de licença compulsória que, no Brasil, na maioria das vezes, estaria condicionada à disponibilidade de versão genérica no mercado internacional para suprir as necessidades até que a produção local garanta o abastecimento. Isso porque o parque industrial químico e farmacêuticos local teria que ter um período para iniciar a produção.

Alternativamente, o uso prévio da flexibilidade do TRIPS, conhecida como exceção Bolar,^2^ possibilitaria desenvolvimento do produto ainda durante a vigência da patente, facilitaria a emissão da licença compulsória e o processo de negociação. Permitiria ainda produzir mais rapidamente o produto no local, além de estimar o custo de produção. Essas opções parecem não ter sido consideradas entre as estratégias para aumentar o poder de barganha do governo de redução de preços do ATV.

Uma alternativa utilizada pelo governo brasileiro, desde 2008, quando lançada a Política de Desenvolvimento Produtivo e, posteriormente, o Plano Brasil Maior em 2011, foi o estímulo à produção local de medicamentos adotados pelo SUS^5,23^ por meio de PDP.

A adoção de PDP para medicamentos incluiu produtos em situação de monopólio ou não. A transferência da tecnologia podia envolver um detentor da tecnologia, um produtor nacional privado para produção do insumo farmacêutico ativo e um produtor público para a produção do produto final, que, por sua vez, pode comercializar o produto exclusivamente no setor público. Em síntese, o poder de compra do governo passou a ser usado como indutor da produção local de medicamentos e de farmoquímicos porque garantiu mercado a um produtor preferencial para produtos definidos como prioritários, adotados pelo MS (Portarias MS 978/2008, 1.284/2010 e, posteriormente, 3.089/2013).

Embora promover a produção local e reduzir a dependência externa do Brasil no setor farmacêutico tenham sido objetivos da PDP, o monopólio criado pela garantia de mercado a um produtor preferencial pode ter impacto negativo sobre os preços.^11,16^ Isso porque cria-se monopólio para os produtos não patenteados e mantém-se e reforça-se o monopólio para aqueles que são patenteados.

Algumas PDP vêm sendo anunciadas desde 2009, porém, os objetivos específicos sobre seu uso foram inicialmente definidos em 2012 (Portaria MS 837/2012). Os objetivos incluíam: racionalização do poder de compra do governo; maior cooperação no desenvolvimento tecnológico e intercâmbio de conhecimento entre produtores públicos e privados; produção local de medicamentos estratégicos, caros e prioritários para assegurar a disponibilidade; e negociações progressivas para reduções significativas de preços.

Não estavam detalhados, entre os objetivos específicos, como seria a cooperação no desenvolvimento tecnológico e no intercâmbio de conhecimentos. A incorporação de tecnologia de forma isolada não é suficiente para aumentar o poder governamental de barganha. Se não houver previsão de investimentos em capacitação tecnológica e treinamento de força de trabalho, a transferência de tecnologia de fato não ocorrerá.^8^ Sem garantia de acumulação e capacitação tecnológica, as empresas locais não estarão habilitadas a colaborar no desenvolvimento de novas e melhores tecnologias.^15,j^

Além disso, somente a acumulação tecnológica permitirá negociar contratos de transferência de tecnologia em melhores condições de assimetria de informação entre comprador e vendedor da tecnologia;^j^ estimar o custo de produção de medicamentos conhecendo a margem de comercialização das empresas; e aumentar o potencial de emitir licenças compulsórias através da produção local, caso as negociações não sejam vantajosas.

O governo brasileiro anunciou a intenção de implementar 10 PDP para produção local de ARV entre 2009 e final de 2012.^k^ Entre essas, figura a PDP do atazanavir, assinada em 2011 entre BMS e Farmanguinhos – Instituto de Tecnologia em Fármacos da Fundação Oswaldo Cruz (produtor público), para licença voluntária da patente vigente e transferência de tecnologia do insumo farmacêutico ativo e das formas farmacêuticas em cápsulas de 200 mg e 300 mg.^l^ O presente estudo focaliza aspectos desse Acordo relacionados a preços e possível fortalecimento do monopólio.

O Acordo estabelece entre os resultados previstos a redução de 5,0% ao ano ao longo de cinco anos para as duas apresentações. Assumindo que ele entrou em vigor em janeiro de 2012 e que o preço de partida foi aquele pago em 2011, registrado no SIASG, foi possível estimar os 25,0% de redução até 2016 para as duas concentrações pela variação nos preços unitários (Figuras 2 e 3). Apesar de os preços para 2012 e 2013 apontarem consistência com a redução estimada quando ajustados pelo Índice de Preços ao Consumidor Amplo, em valores nominais, os preços são praticamente constantes para as duas apresentações entre 2011 e 2013. Na prática, a redução reflete mais os ajustes ao índice de inflação.

Reduções mais significativas de preço foram alcançadas no período anterior à assinatura da PDP. A redução para a cápsula de 200 mg foi de 37,6% entre 2009 e 2011. Para a cápsula de 300 mg, a redução foi de 49,0% no mesmo período.

Estudos sobre licenças voluntárias de ARV^m,n^ apontam que o detentor da tecnologia negocia cláusulas restritivas que podem limitar a possibilidade de futuras reduções de preços. O Acordo entre Farmanguinhos e BMS explicitamente impede a produção de outras apresentações ou de combinações em doses fixas, que não sejam as cápsulas de 200 mg e 300 mg. No entanto, uma combinação em dose fixa de atazanavir e ritonavir em comprimido (termoestável) foi incluída no protocolo terapêutico da OMS em 2013. Se essa combinação se tornar a opção preferencial e for adotada no consenso nacional de HIV, as cápsulas produzidas por Farmanguinhos estarão obsoletas.

Alguns termos do Acordo podem limitar a possibilidade de futuras reduções de preços. Farmanguinhos está obrigado a comprar da BMS 100% da demanda do MS nos três primeiros anos após a concessão do registro sanitário. Nos quarto e quinto anos, a BMS continuará responsável por 50,0% da demanda do MS. Ou seja, caso houvesse algum atraso no processo de transferência de tecnologia a Farmanguinhos ou no registro sanitário, a BMS estaria assegurando mercado mesmo após expiração da patente. A patente está prestes a expirar, logo a licença voluntária representa uma oportunidade para a BMS explorar o valor comercial restante da patente.

No final de 2013, o MPP negociou uma licença voluntária para o atazanavir com a BMS contendo cláusulas menos restritivas do que aquelas previstas no Acordo com Farmanguinhos, como a possibilidade de o licenciado produzir quaisquer tipos de apresentações e combinações em doses fixas.^o^

Algumas questões merecem análise mais aprofundada. Uma delas é se a PDP se constitui como a estratégia mais adequada para superar a barreira patentária e alcançar redução de preços. O governo, ao ficar aprisionado por um Acordo, abre mão da possibilidade de adotar outras estratégias antimonopólio, caso o ambiente nacional e internacional torne-se mais concorrencial.

A segunda é a interface entre produção local e acesso. Revisão da literatura internacional^p^ explora essa relação e mostra que para alguns casos é duvidoso o benefício no curto prazo conferido pela produção local na economia de recursos, exemplificando países como o Brasil. Por um lado, poder-se-ia argumentar que esta produção pode assegurar a disponibilidade interna do medicamento no mercado nacional. Na prática, essa disponibilidade já existe, pois o atazanavir, assim como outros produtos prioritários para PDP, é importado e representa um mercado público atrativo o suficiente para que as empresas multinacionais não deixem de fornecer seus produtos ao Brasil. Por outro, a produção local poderia melhorar o poder de barganha das compras públicas de medicamentos em situação de monopólio, além de representar possibilidade de suprimento estratégico na presença de dificuldades de importação.

O volume da aquisição e a compra centralizada de ARV patenteado parecem ter pouco ou nenhum efeito no preço. O caso mostra a complexidade e as dificuldades enfrentadas por gestores em saúde para reduzir o preço de medicamentos em um ambiente de concorrência limitada. É necessária uma abordagem multifacetada para alcançar reduções de preços. O uso das flexibilidades do TRIPS para enfrentar a barreira patentária como oposições de patentes, exceção Bolar e licenças compulsórias, que não foram adotadas neste caso, deveriam também ser utilizadas como instrumento de fortalecimento das negociações de preços.

O Acordo, que estabelece a PDP do atazanavir, levanta várias questões em relação ao uso somente dessas parcerias para reduzir preços e enfrentar a barreira patentária. Os termos nos quais o Brasil entra na PDP podem inibir as empresas a aderirem às licenças voluntárias internacionais realizadas pelo MPP. São necessárias mais investigações para entender como essas cláusulas foram negociadas e quais estratégias poderiam ter sido exploradas para ampliar a capacidade de o governo obter cláusulas mais favoráveis com o detentor da tecnologia.

Se o governo seguir com a opção de fortalecimento da produção local de medicamentos por meio da estratégia da PDP, deverá considerar também investimentos em P&D que estimulem a acumulação tecnológica. Isso fortaleceria o poder de barganha do governo em negociar preços de medicamentos em situação de monopólio e contribuiria de fato com a sustentabilidade da política de acesso do SUS.

## Footnotes

a Santos RSL. Sustentabilidade do Programa Nacional de DST/aids: análise da capacidade de oferta e preços dos medicamentos antirretrovirais [dissertação]. Rio de Janeiro (RJ): Instituto de Economia da Universidade Federal do Rio de Janeiro; 2010.

b Medicines Patent Pool [Internet]. About the MPP. [citado 2014 fev 23]. Disponível em: http://www.medicinespatentpool.org/about

c Médecins San Frontières, Access Campaign. Untangling the web of antirretroviral price reductions [internet]. 17.ed. Geneva: MSF; 2014 [citado 2014 nov 15]. Disponível em: https://www.msfaccess.org/sites/default/files/MSF_UTW_17th_Edition_4_b.pdf

d Villardi P. Panorama do status patentário e registro sanitário dos medicamentos antirretrovirais no Brasil: implicações para o acesso e a política industrial no Brasil. Rio de Janeiro (RJ): Associação Brasileira Interdisciplinar de Aids; 2012 [citado 2014 jun 12]. Disponível em: http://www.deolhonaspatentes.org.br/media/file/Publica%C3%A7%C3%B5es/Publica%C3%A7%C3%A3o%20Pedro_Final_23OUT.pdf

e Scheffer MC. Aids, tecnologia e acesso sustentável a medicamentos: a incorporação dos antirretrovirais no Sistema Único de Saúde [tese]. São Paulo (SP): Faculdade de Medicina da Universidade de São Paulo; 2008.

f Médecins Sans Frontières, Access Campaign. Untangling the web of antiretroviral price reductions. 13.ed. Geneva; 2010 [citado 2013 abr 25]. Disponível em: http://d2pd3b5abq75bb.cloudfront.net/2012/07/16/14/39/31/171/UTW_13_ENG_Jul2010.pdf

g World Health Organization. How to develop and implement a national drug policy: updates and replaces: guidelines for developing national drug policies, 1988. 2.ed. Geneva; 2013 [citado 2014 jan 15]. Disponível em: http://apps.who.int/medicinedocs/pdf/s2283e/s2283e.pdf

h Joint United Nations Programme on HIV/AIDS. Doha+10 trips flexibilities and access to antiretroviral therapy: lessons from the past, opportunities for the future. Geneva: Unaids; 2011 [citado 2014 jan 20]. Disponível em: http://www.unaids.org/en/media/unaids/contentassets/documents/unaidspublication/2011/JC2260_DOHA+10TRIPS_en.pdf

i United Nations Conference on Trade and Development (UNCTAD). Using intellectual property rights to stimulate pharmaceutical production in developing countries: a reference guide. Geneva: United Nations; 2011 [citado 2014 jan 20]. Disponível em: http://unctad.org/en/Docs/diaepcb2009d19_en.pdf

j Foray D. Technology transfer in the TRIPS age: the need for new types of partnerships between the least developed and most advanced economies. Geneva: ICTSD; 2009 [citado 2014 jan 20]. (ICTSD Intellectual Property and Sustainable Development Series; issue paper nº 23). Disponível em: http://ictsd.org/downloads/2012/02/technology-transfer-in-the-trips-age.pdf

k Ministério da Saúde. Propostas de Projetos de Parcerias para o Desenvolvimento Produtivo (PDP) aprovadas de 2009 a 2014. Brasília (DF); 2015. [citado 2015 out 26]. Disponível em: http://portalsaude.saude.gov.br/images/pdf/2015/outubro/01/Propostas-de-projeto-de-PDP-aprovadas-de-2009-a-2014-01-10-2015.pdf

l Agência Fiocruz de Notícias. Fundação assina acordo para produção do medicamento Atazanavir. Rio de Janeiro; Fiocruz; 2011 [citado 2013 mar 26]. Disponível em: http://www.agencia.fiocruz.br/funda%C3%A7%C3%A3o-assina-acordo-para-produ%C3%A7%C3%A3o-do-medicamento-atazanavir

m Park C, Moon S, Burrone E, Boulet P, Juneja S, ‘t Hoen E, et al. Voluntary licensing: an analysis of current practices and key provisions in antiretroviral voluntary licences. Geneva: Medicines Patent Pool; 2012 [citado 2013 out 27]. Disponível em: http://www.medicinespatentpool.org/wp-content/uploads/Current-Practice-and-Key-Provisions-in-ARV-VLs.pdf

n Médecins Sans Frontières, Access Campaign. Barriers to accessing generics: the restrictions posed by certain voluntary license agreements. Geneva; 2012 [citado 2015 out 26]. Disponível em: http://www.msfaccess.org/sites/default/files/MSF_assets/HIV_AIDS/Docs/AIDS_report_UTW15_ENG_2012.pdf

o Medicines Patent Pool. License and technology transfer agreement. Geneva; 2013 [citado 2014 jan 10]. Disponível em: http://www.medicinespatentpool.org/wp-content/uploads/MPP-License-and-technology-transfer-agreement-Signed.pdf

p World Health Organization. Local production for access to medical products: developing a framework to improve public health. Geneva; 2011 [citado 2014 jan 10]. Disponível em http://www.who.int/phi/publications/Local_Production_Policy_Framework.pdf

## DESTAQUES

A pesquisa estuda, usando como exemplo o caso do atazanavir, como o Brasil tem enfrentado a alta dos preços de medicamentos em situação de monopólio.

O atazanavir é um inibidor de protease cujos gastos, crescentes ao longo do tempo, favoreceram sua inclusão em uma Parceria de Desenvolvimento Produtivo pelo Ministério da Saúde. Como o preço desse tipo de medicamento é pouco sensível ao volume de compra, a Parceria de Desenvolvimento Produtivo passa a ser importante estratégia de redução de preços, uma vez que o poder de barganha do monopolista diminui diante da capacitação tecnológica e industrial do País para fabricar o medicamento.

Os preços do atazanavir no Brasil são mais altos do que os das versões genéricas disponíveis e do que o menor preço do fabricante. A redução esperada, de 5,0% ao ano, não foi igual para todas as apresentações do atazanavir. Para a cápsula de 200 mg, as reduções observadas foram menores do que a redução estimada e para a cápsula de 300 mg e os valores pagos nos dois primeiros anos, após a Parceria de Desenvolvimento Produtivo, foram próximos aos valores estimados.

Para alcance de redução de preços de produtos em situação de monopólio, recomenda-se a aplicação de uma abordagem multifacetada, que inclua não só o instrumento da Parceria de Desenvolvimento Produtivo, mas também o uso de flexibilidades do Acordo sobre os Aspectos dos Direitos de Propriedade Intelectual Relacionados ao Comércio de proteção da saúde pública, como licença compulsória, exceção Bolar e oposição de patentes.

Rita de Cássia Barradas Barata

Editora Científica
